# French Translation and Validation of Three Scales Evaluating Stigma in Mental Health

**DOI:** 10.3389/fpsyt.2017.00290

**Published:** 2017-12-18

**Authors:** Carla Garcia, Philippe Golay, Jérôme Favrod, Charles Bonsack

**Affiliations:** ^1^Community Psychiatry Unit, Department of Psychiatry, Centre Hospitalier Universitaire Vaudois, Lausanne, Switzerland; ^2^School of Nursing Sciences La Source, University of Applied Sciences and Arts of Western Switzerland, Lausanne, Switzerland

**Keywords:** stigma, mental illness, discrimination, validity, reliability, confirmatory factor analysis

## Abstract

**Objective:**

The concept of stigma refers to problems of knowledge (ignorance), attitudes (prejudice), and behavior (discrimination). Stigma may hinder access to care, housing, and work. In the context of implementation of programs such as “housing first” or “individual placement and support” in French speaking regions, validated instruments measuring stigma are necessary. “Attitudes to Mental Illness 2011” is a questionnaire that includes three scales measuring stigma through these three dimensions. This study aimed to translate, adapt, and validate these three scales in French.

**Methods:**

The “Attitudes to Mental Illness 2011” questionnaire was translated into French and back-translated into English by an expert. Two hundred and sixty-eight nursing students completed the questionnaire. Content validity, face validity, internal validity, and convergent validity were assessed. Long-term reliability was also estimated over a three-month period.

**Results:**

Experts and participants found that the questionnaire’s content validity and face validity were appropriate. The internal validities of the three scales were also considered adequate. Convergent validity indicated that the scales did indeed measure what they were supposed to. Long-term stability estimates were moderate; this pattern of results suggested that the construct targeted by the three scales is adequately measured but does not necessarily represent stable and enduring traits.

**Conclusion:**

Because of their good psychometric properties, these three scales can be used in French, either separately, to measure one specific dimension of stigma, or together, to assess stigma in its three dimensions. This would seem of paramount importance in evaluating campaigns against stigma since it allows measures to be adapted according to campaign goals and the target population.

## Introduction

Around the world, the stigma of mental illness is a very common problem, one which persists over time and has a significant impact on public health. Stigma can be seen as an umbrella term made up of three dimensions: problems of knowledge (ignorance), problems of attitudes (prejudice), and problems of behavior (discrimination) (1). The literature shows a direct relationship between these three dimensions and the process of recovery from a psychiatric disorder: low levels of knowledge, stigmatizing attitudes and discriminatory behavior are associated with social exclusion and lower rates of help-seeking and medication compliance, all of which hinder care and treatment and, therefore, prevent recovery (2–5).

As Link et al. stated, “Essential to the scientific understanding of stigma is our capacity to observe and measure it” (6). A variety of different scales has been created to explore stigma against people with mental illness (7). Some of these scales concentrate on one or two of the three dimensions (generally attitudes and behavior) that make up the broader term of stigma proposed by Thornicroft; most of them use clinical vignettes, which reduces the scale’s margin of representation (8).

The present study used the Attitudes to Mental Illness 2011 questionnaire, created as a part of the UK’s Time to Change Programme (TTC) 2008–2012 anti-stigma campaign. The questionnaire was composed of a shortened list of items from the Community Attitudes toward the Mentally Ill (CAMI) (9) scale and the Opinions about Mental Illness Scale in order to measure prejudice. Mental Health Knowledge Schedule (MAKS) (10) and the Reported and Intended Behavior Scale (RIBS) (11) were then developed to assess knowledge and behavior, in order to measure every dimension of stigma as defined by Thornicroft (12). In previous studies these three scales showed to be sensitive to anti-stigma centered actions (13, 14). Further details on these three scales are provided in the Section “Materials and Methods” of this article.

The Attitudes to Mental Illness 2011 questionnaire was chosen firstly because it does not use clinical vignettes, thus widening the field of possible representations and, second, because this questionnaire faithfully reflects Thornicroft’s three dimension concept of stigma (knowledge, attitudes, and behavior).

To our knowledge, validated instruments measuring public stigma are highly needed in French. Recently, the CAMI scale was used to study an anti-stigma campaign in France that measured changes on the opinions about mental illness in French health professionals after receiving a short training intervention program. This study showed no publication of psychometric data (14). Another recent study validated in French the Stigma scale, which measures perceived stigma. The French scale showed good psychometric properties and an abbreviated version was also developed with satisfactory psychometrics results (15).

This study aimed to translate, adapt, and validate the three scales included in the Attitudes to Mental Illness 2011 questionnaire into French (MAKS, CAMI, and RIBS). To do this, we had to investigate the internal validity of the MAKS, CAMI, and RIBS scales in French and verify their long-term stability, face validity, and convergent validity.

## Materials and Methods

### Participants

Participants were students from La Source School of Nursing Sciences at the University of Applied Sciences and Arts of Western Switzerland (HES La Source). Nurse students were supposed to be interested in the subject of mental illness stigma, while express a wide variety of problems of knowledge, prejudice, and behavior such as professionals of care (16). A poor French language skill was the only exclusion criterion, as this may have hindered a participant’s ability to accurately respond to questions. The authors of this article, using Sphinx software, developed an electronic version of the questionnaire. All the students of the HES La Source (*n* = 750) were invited to answer the questionnaire *via* email. The confirmatory factor analysis (CFA) is the analysis that is the most demanding with regard to sample size [for the test–retest reliability and the concurrent reliability which are based on Pearson’s R the required sample size to detect a correlation of 0.4 with 0.95 power and alpha set to 0.05 is relatively low (*N* = 75)]. It is difficult to estimate precisely the needed sample size for CFA because it is a function of several factors. Sample less than 100 could lead to increased over-rejection rates for indices of goodness of fit such as the root mean square error of approximation (RMSEA). Based on a lot of similar studies, we aimed for a sample size of 250 which appeared sufficient given the relatively low complexity of the models. To assess test–retest reliability, a second assessment was made 3 months after the first.

### Measures

We adapted and validated French versions of the three scales contained in the Attitudes to Mental Illness 2011 questionnaire. Each scale measures a different dimension of the concept of stigma (knowledge, attitudes, and behavior).

#### The MAKS (12 Items) (10)

This scale consists of two parts. Part A includes six items covering areas of knowledge related to the stigma attached to mental health (help-seeking, acknowledgment, support, work, treatment, and recovery); Part B includes six items that examine the classification of different conditions as mental illness. The items are coded on an ordinal scale (1–5). Items which the respondent strongly agrees with score 5 points; 1 point reflects a response to which the respondent strongly disagrees. The total score is calculated by adding the points obtained for each of the 12 items. Two subtotals (Parts A and B) can also be computed. In previous studies, MASK showed an overall test–retest reliability of 0.71 using Lin’s concordance statistic. The overall internal consistency among items 1 to 6 was moderate (0.65) (10). Because MAKS is designed to measure a heterogeneous group of items, high internal consistency is not expected; respondents’ knowledge (whether good or bad) may only be related to specific areas of mental health. Higher total scores correspond to greater knowledge.

#### The UK Department of Health’s Community Attitudes toward the Mentally Ill Questionnaire (27 Items) (9)

This scale consists of the CAMI scale (26 items), plus one added item on job-related attitudes. The original validation of this questionnaire involved a model with four factors: Authoritarianism, Benevolence, Social restriction, and Community mental health ideology. The answers are coded on a Likert scale from 1 (strongly disagree) to 5 (strongly agree). A total score and four subtotals can be calculated. A higher total score indicates less stigmatizing attitudes. In previous studies, the CAMI scale showed a satisfactory overall internal consistency using Cronbach’s α (0.87) (9).

#### The RIBS (8 Items) (11)

This scale’s eight items come in two groups of four. The first group focuses on behavior reported in past or present experiences regarding the following areas: live with, work with, live nearby, or have a relationship with a person with a mental health problem. The second group focuses on future intentions to establish contact with people with a mental health problem in the same areas as described above. Because items 1–4 only calculate the prevalence of behaviors which respondents may or not have had, no final scale points are given for them. The final total score is calculated by adding the points obtained for items 5–8, each coded on an ordinal scale (1–5 points). “Do not know” is coded as neutral (i.e., 3). The total score is calculated so that high values correspond to more favorable expected behaviors. In previous studies, RIBS showed an overall test–retest reliability of 0.75. The overall internal consistency, based on Cronbach’s alpha among items 5–8 was 0.85. The RIBS demonstrated overall moderate/substantial test–retest reliability and substantial internal consistency (11).

### Procedure

The Attitudes to Mental Illness 2011 questionnaire was obtained from one of its authors (Sara Evans-Lacko). It was first translated into French by two authors of the present study, Carla Garcia and Jérôme Favrod. The versions were compared and adjusted to best match the meaning of the original scale. The resulting version was then back-translated into English by a third English native speaker who was blind to the original version. This English version was sent to Sara Evans-Lacko, who agreed that it captured the essential nuances of the original version. To assess the content validity of the questionnaire’s French version, it was sent to a variety of experts (e.g., nurses and psychologists), before sending it to the population target in this study. They agreed that the questions asked were coherent. To assess the face validity with the participants, three questions, each one measuring one of the three dimensions of stigma, asked for the scale to be rated from 0 to 100.

### Internal Validity

In order to evaluate the internal validity, we tested the original CAMI four-factor model (13), which included an Authoritarianism factor (items 1–7), a Benevolence factor (items 8–14), a Social restrictiveness factor (items 15–21), and a Community mental health ideology factor (items 22–26). This was compared to a single-factor model which corresponded to the CAMI total score. Internal validity for the proposed one-factor model of RIBS was estimated using only items 5–8, because items 1–4 are used to assess prevalence and do not contribute to the total score (11). Internal validity for MAKS tested the original two-factor solution (10), including a Mental health knowledge factor (items 1–6) and a Mental-illness condition knowledge factor (items 7–12). This was compared with a simpler one-factor alternative.

### Convergent Validity

To estimate convergent validity, several indicators were used to study the relationship between the scores. We hypothesized that MAKS scores were positively related to both RIBS and CAMI scores. We also asked the participants to answer three questions, each one measuring one of the three dimensions of knowledge, attitudes, and behavior, on a scale (control scale) rated from 0 to 100. We hypothesized that each scale would be positively related with the control scale.

### Reliability

A test–retest approach, with a 3-month interval between the assessments, was used to estimate the long-term reliability of the test scores.

### Ethical Considerations

As the sample consisted of a non-clinical population, this study required no ethical approvals, in accordance with national and institutional guidelines. The request for consent to participate was made in the communication which comprehensively explained the nature and purpose of the study. A positive answer signaled the respondent’s agreement to participate in the study. Participation was anonymized and each participant was attributed a code.

### Statistical Analysis

All statistical tests were two-tailed and a significance level was set at *p* = 0.05.

#### Internal Validity

All the reverse-scored items were re-coded prior to data analysis. For CFA, item data were treated as categorical ordinals and the models were evaluated using a robust weighted least squares mean- and variance-adjusted estimation. For CAMI, the original four-factor model was estimated first. This model was compared to a more parsimonious model including one-factor. The one-factor model was estimated for RIBS. For the MAKS, the original two-factor solution was compared to the single-factor alternative. Several indicators of model fit were used, such as the RMSEA, the comparison fit index (CFI), and the Tucker–Lewis fit index (TLI). A RMSEA less than 0.06, and a CFI and TLI larger than 0.95 are interpreted as good fits, whereas values of RMSEA ≤0.08 and CFI/TLI ≥0.90 are often considered as acceptable fits (17). The RMSEA has been found to falsely reject properly specified models with a small number of degrees of freedom (18). Because the MAKS scale only includes four items, our model evaluation was mainly based on the CFI and TLI coefficients. Furthermore, the interpretation of overall fit indexes in models with ordered categorical indicators is not as well established as it is with continuous indicators (19). Although simulation studies suggest that these cut-off values work reasonably well with categorical outcomes (20), the exact cut-off scores may not apply perfectly in the context of the present study. For this reason, alternative models were compared using a robust chi-square test using the DIFFTEST procedure featured in the Mplus statistical package, version 7.4.

#### Convergent Validity

Convergent validity was assessed using Pearson correlation coefficients. Reliability and convergent validity analyses were performed using IBM SPSS, version 22.

#### Reliability

The long-term stability of the scores was investigated by carrying out a second assessment after 3 months. The relative test–retest reliability was estimated using both the Pearson and intra-class correlation coefficients, using a two-way random-effects model and the absolute agreement definition (ICC (2,1)). For the computation of total scores, given that at least 50% of items were answered, missing data were replaced by individual mean values.

## Results

### Participants

268 students fully answered the questionnaire. Sixty-one participants answered to a second test–retest assessment.

### Face Validity

The face validity of the CAMI was rated at an average of 61.2 (±26.0) out of 100. The median estimate was 65. Face validity estimates for the RIBS and MAKS were very similar (mean 62.3 ± 25.7, median = 65, and mean 58.0 ± 26.6, median = 60, respectively).

### Internal Validity

**Figure 1:**
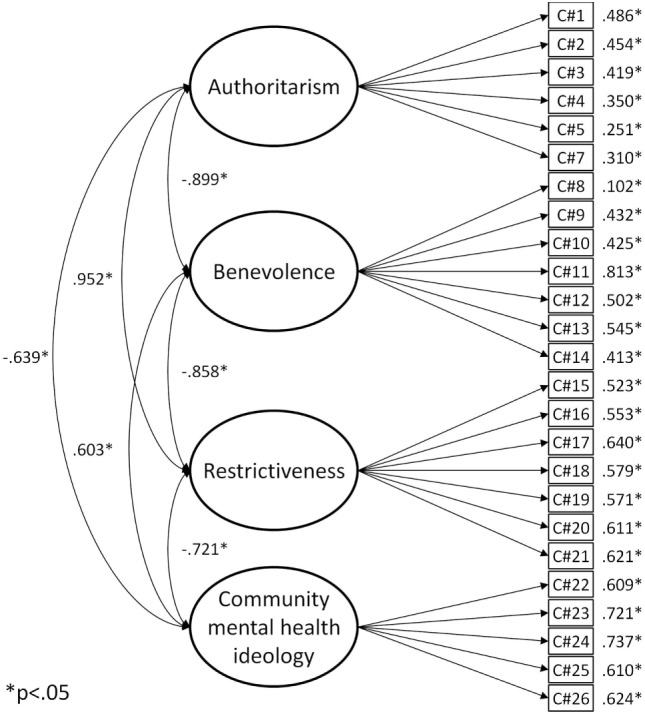
CAMI modified four-factor model.

As shown in Table 1, the RMSEA coefficient assessment of the four-factor CAMI model’s fit was excellent; however, its fit was less satisfactory according to its CFI and TLI values. Interestingly, the loading between item 6 and the Authoritarianism factor was not significant. A modified version, discarding this problematic item yielded a similar fit but all expected factor loadings were supported (see Figure 1). Factor correlations were very high overall, suggesting that all items could potentially be explained by one dimension. On the basis of CAMI’s 25 items, an alternative, simpler, one-factor model was estimated and compared to the four-factor version. Model fit seemed slightly less adequate than the four-factor solution. Because these models were statistically nested, they could be compared using a robust chi-square difference test. The result confirmed that the four-factor model had a significantly better fit than the one-factor model and should, therefore, be preferred (Δχ^2^ = 40.982, Δdf = 6, *p* < 0.001).

**Table 1:** Comparisons of model fit for the three stigma scales (*N* = 268).

Model	χ^2^	Df	*p*-Value	RMSEA	CFI	TLI
**Community attitudes toward the mentally ill scale**																											
Four-factor model	442.253	293	<0.001	0.044	0.869	0.855
Modified four-factor model^a^	403.729	269	<0.001	0.043	0.882	0.868
One-factor model^a^	442.016	275	<0.001	0.048	0.853	0.840
**Reported and Intended Behavior Scale**						
One-factor model	6.575	2	0.037	0.092	0.985	0.954
**Mental Health Knowledge Schedule scale**						
Two-factor model	212.844	53	<0.001	0.106	0.790	0.739
Modified two-factor model^b^	43.249	19	0.001	0.069	0.947	0.923
One-factor model^b^	74.278	20	<0.001	0.101	0.882	0.835

*^a^With item 6 removed*.

*^b^With items 1, 6, 8, and 12 removed*.

*df, degree of freedom; RMSEA, root mean square error of approximation; CFI, comparative fit index; TLI, Tucker–Lewis index*.

**Figure 2:**
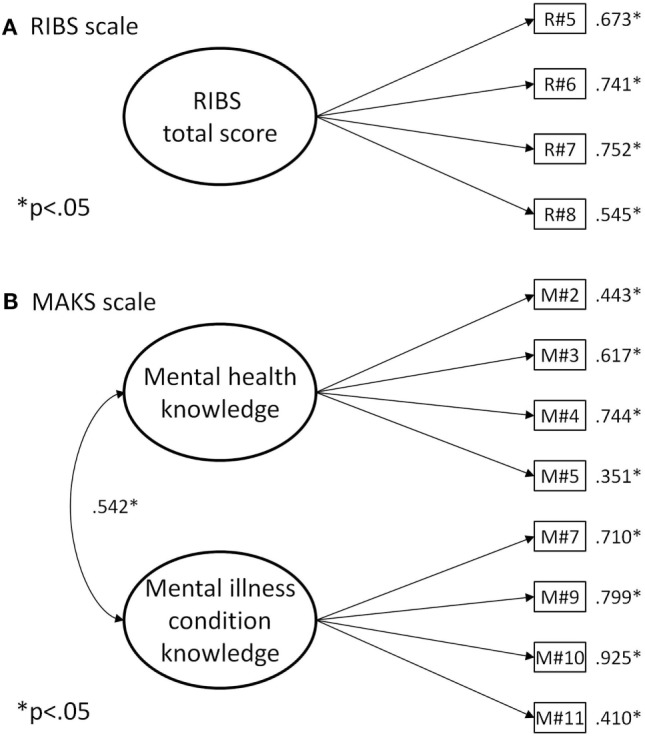
**(A)** Reported and Intended Behavior Scale (RIBS), **(B)** Mental Health Knowledge Schedule (MAKS) scale.

As shown in Table 1, the model fits of the RIBS scale were excellent according to the CFI and the TLI coefficients and all the factor loadings were supported (see Figure 2A).

As shown in Table 1, the model fit of the MAKS two-factor model was poor. Four items were problematic: the factor loadings of items 1 and 6 were not statistically significant, and the factor loadings of items 8 and 12 were negative. The model fit of the two-factor model, without these four items, was strongly improved and could be considered as adequate (see Figure 2B). Because correlation between the two factors was substantial (*r* = 0.542, *p* < 0.001) a more parsimonious model was estimated, involving only one general factor. The model fit was poor, and the results of the robust chi-square difference tests between these two nested models confirmed that the two-factor version should be preferred (Δχ^2^ = 23.427, Δdf = 1, *p* < 0.001).

### Convergent Validity

Convergent validity evidence is presented in Table 2. All the CAMI subscores were related to the Right to individual housing control scale. They were also related to the RIBS and MAKS scores. The RIBS score was also significantly related to the Knowledge of mental health control scale, and it was related to both the CAMI and MAKS scores. Finally, the MAKS subscores were related to the Ability to integrate community life control scale. In summary, all the correlations that we expected to observe occurred in the direction hypothesized and were statistically significant.

**Table 2:** Convergent validity of the three stigma scales.

	1	2	3	4	5	6	7	8	9	10	11	12
1. Community attitudes toward the mentally ill (CAMI) Authoritarianism	–											
2. CAMI Benevolence	−0.302*	–										
3. CAMI Social restrictiveness	0.344*	−0.284*	–									
4. CAMI Community mental health ideology	−0.303*	0.289*	0.390*	–								
5. CAMI Overall score	0.718*	−0.632*	0.727*	−0.715*	–							
6. Right to individual housing control scale	−0.374*	0.205*	−0.403*	0.247*	−0.444*	–						
7. Reported and Intended Behavior Scale overall score	−0.292*	0.182*	−0.334*	0.419*	−0.447*	0.295*	–					
8. Knowledge of mental health control scale	−0.352*	0.057	−0.237*	0.206*	−0.322*	0.309*	0.192*	–				
9. Mental Health Knowledge Schedule mental health knowledge	−0.273*	0.195*	−0.184*	0.255*	−0.332*	0.254*	0.184*	0.428*	–			
10. MAKS mental illness condition knowledge	−0.194*	0.115	−0.107	0.033	−0.160*	0.160*	0.126*	0.229*	0.201*	–		
11. MAKS overall score	−0.314*	0.180*	−0.163*	0.178*	−0.305*	0.237*	0.205*	0.414*	0.793*	0.756*	–	
12. Ability to integrate community life control scale	−0.350*	0.208*	−0.311*	0.378*	−0.450*	0.502*	0.313*	0.349*	0.208*	0.168*	0.237*	–

*p < 0.05.

### Reliability

Long-term stability estimates, shown in Table 3, suggested only moderate long-term stability over the 3-month period.

**Table 3:** Long-term stability of the three stigma scales.

	*N*	Pearson’s r	ICC (2,1)	95% C.I. ICC (2,1)
**Community attitudes toward the mentally ill scale**				
Authoritarianism	61	0.632*	0.578*	0.358–0.732
Benevolence	61	0.391*	0.391*	0.156–0.584
Social restrictiveness	61	0.625*	0.624*	0.444–0.755
Community mental health ideology	60	0.633*	0.636*	0.456–0.765
Overall score	61	0.804*	0.799*	0.687–0.875
**Reported and Intended Behavior Scale**				
Overall score	56	0.434*	0.435*	0.195–0.626
**Mental Health Knowledge Schedule scale**				
Mental health knowledge items	52	0.489*	0.485*	0.248–0.667
Mental illness condition knowledge items	59	0.675*	0.671*	0.504–0.790
Overall score	59	0.720*	0.713*	0.561–0.819

*p < 0.05. C.I., confidence interval. ICC (2,1), intra-class correlation coefficient using a 2-way random-effects model and the absolute agreement definition.

## Discussion

Face validity estimates indicated that participants scored all three scales in the upper-middle range. This could suggest that most participants considered the scales to be adequate and that they measure what they are supposed to measure, i.e., the three domains of stigma (knowledge, attitudes, and behavior).

Results from the CFA indicated that an adequate four-factor model should be favored for the French version of CAMI as in the original version (9). The expected structure was also replicated in the French version of RIBS, which was the same as the original created by S. Evans-Lacko et al. (11) For the MAKS scale, all the problematic items proved to be reverse-coded items. Indeed, we had hypothesized that they would be less well understood by a portion of the participants. Therefore, an adapted version of the MAKS scale, without the reverse-coded items, might be a more successful proposition for use in future studies in French speaking regions. CFA revealed that every item in each of the three adapted scales contributed significantly to its respective scale’s dimension, and that the internal validity of each scale could be considered as adequate, as it was for the original scales (9–11). Furthermore, convergent validity estimates confirmed the relevance of all three French versions of the scales. Indeed, the present study’s results suggest that all three French versions of these scales do in fact measure what they are supposed to. Long-term stability, however, was only moderate, similar findings was shown with the original models of RIBS and MAKS (10, 11). Given the adequate internal and convergent validities found, this pattern of results suggests that the construct targeted by these three scales are adequately measured but do not represent stable and enduring traits. As found in previous studies (9–11), the three dimensions of stigma are probably subject to change over a relatively short time frame and should, thus, be assessed regularly. This is an important point since very stable traits (e.g., intelligence or personality) could be more difficult to target and change using psychosocial interventions.

Regarding the present study’s limitations, it should be considered that the sample was composed of nursing students, participants who may have different views about the stigmatization of mental health problems than the general population. In our opinion, however, there are no theoretical reasons to expect a significant bias in our results because all the analyses were based on covariances and not on average levels. The range reduction in the observed scores may have underestimated correlations which might have been higher in a less homogeneous sample. Nevertheless, nurse students may be more motivated to answer than general population; this could represent a moderate bias in the feasibility and acceptability of the scales.

With the French validation of the Attitudes to Mental Illness 2011 questionnaire, we created a French scale that measures public stigma in a three dimension approach. This tool can be used in the future to help the implementation of programs such as “housing first” or “individual placement and support” in French speaking regions, through the recognition of possible barriers linked to stigma. A strong point of the Attitudes to Mental Illness 2011 questionnaire, translated into French and tested in this study, is that its component scales can be used separately to measure one specific dimension of stigma, or together, to assess stigma in its three dimensions of knowledge, attitudes and behavior. This aspect would be of paramount importance in the evaluation of anti-stigma campaigns since it would allow the measurement scales to be adapted according to the campaign goals and the target population.

The original structure of the Community Attitudes toward the Mentally Ill scale, without item 6, should be proposed as the French version. The French version of the RIBS could replicate the original structure. A French version of the MAKS should be adapted to exclude the reverse-coded items (see Table 4).

**Table 4:** French version of the community attitudes toward the mentally ill (CAMI) scale, Mental Health Knowledge Schedule (MAKS) scale, and Reported and Intended Behavior Scale (RIBS) proposed in this study.

Items
**CAMI**
*Facteur 1—Autoritarisme* Une des principales causes de maladie mentale est un manque d’autodiscipline et de volonté. Les personnes avec une maladie mentale ont quelque chose qui les différencie facilement des gens normaux. Aussitôt qu’une personne montre des signes d’un trouble mental, elle devrait être hospitalisée. La maladie mentale est une maladie comme une autre. On devrait moins insister pour protéger le public de personnes avec une maladie mentale. Virtuellement, tout le monde peut développer une maladie mentale. Facteur 2—Bienveillance Les personnes avec des maladies mentales ont été trop longtemps tournées en ridicule. Nous avons besoin d’adopter une attitude beaucoup plus tolérante envers les personnes ayant des maladies mentales dans notre société. Nous avons la responsabilité d’offrir les meilleurs soins possibles aux personnes avec de maladies mentales. Les personnes avec de maladies mentales ne méritent pas notre sympathie. Les personnes avec une maladie mentale sont un fardeau pour la société. Des dépenses d’argent importantes dans les services de santé mentale sont un gaspillage. Il existe assez de services de santé mentale pour les personnes souffrant de maladies mentales. Facteur 3—Restriction Sociale Aucune responsabilité ne devrait être donnée aux personnes avec une maladie mentale. Il serait stupide de se marier avec une personne qui a eu une maladie mentale, même si cette dernière semble complètement rétablie. Je n’aimerais pas habiter à côté de quelqu’un qui a eu une maladie mentale. Toute personne ayant eu une maladie mentale devrait être exclue de la fonction publique. Personne n’a le droit d’exclure de son quartier les personnes avec une maladie mentale. Les personnes avec une maladie mentale sont beaucoup moins dangereuses que ne le suppose la plupart des gens. La majorité des femmes ayant été hospitalisées dans un hôpital psychiatrique sont fiables comme baby-sitters. Facteur 4—idéologie de la communauté sur la santé mentale La meilleure thérapie pour beaucoup de personnes avec une maladie mentale est de faire partie de la communauté normale. Dans la mesure du possible, les services de santé mentale devraient être fournis dans des centres basés dans la communauté. Les résidents n’ont rien à craindre des personnes qui viennent dans leurs quartiers pour obtenir des soins en santé mentale. ça fait peur de penser que des personnes avec des problèmes mentaux puissent vivre dans des quartiers résidentiels. Placer des services de santé mentale dans une zone résidentielle déclasse le quartier.
**MAKS**
*Facteur 1—connaissances en lien avec la stigmatisation liée à la santé mentale* Si un(e) ami(e) a un problème de santé mentale, je sais quel conseil lui donner pour obtenir une aide professionnelle. Les médicaments peuvent être un traitement efficace pour des personnes avec des problèmes de santé mentale. La psychothérapie (ex: thérapie verbale ou conseil) peut être un traitement efficace pour des personnes avec des problèmes de santé mentale. Les personnes avec des graves problèmes de santé mentale peuvent se rétablir complètement. Facteur 2—classification de différentes conditions comme maladie mentale Dépression Schizophrénie Trouble bipolaire (maniaco-dépressif) Dépendance aux drogues
**RIBS**
*Facteur 1—comportements rapportés* Vivez-vous actuellement ou avez-vous vécu une fois avec une personne qui a un problème de santé mentale? Travaillez-vous actuellement ou avez-vous travaillé une fois avec une personne qui a un problème de santé mentale? 2bis. Etudiez-vous actuellement ou avez-vous étudié une fois avec une personne qui a un problème de santé mentale? Avez-vous actuellement ou avez-vous eu une fois un(e) voisin(e) qui a un problème de santé mentale? Avez-vous actuellement ou avez-vous eu une fois un(e) ami(e) proche qui a un problème de santé mentale? Facteur 2—intentions futures Dans le futur, je serais prêt à vivre avec une personne qui a un problème de santé mentale. Dans le futur, je serais prêt à travailler avec une personne qui a un problème de santé mentale. Dans le futur, je serais prêt à habiter à proximité d’une personne qui a un problème de santé mentale. Dans le futur, je serais prêt à conserver mon lien avec un(e) ami(e) qui a développé un problème de santé mentale.

Finally, the changing scores of the three dimensions of knowledge, attitudes, and behavior, measured by these scales over time, give an optimistic outlook on the potential for positive changes resulting from campaigns aiming to reduce the stigmatization of mental health problems.

## Ethics Statement

As the sample consisted of a non-clinical population, this study required no ethical approvals, in accordance with national and institutional guidelines. The request for consent to participate was made in the communication which comprehensively explained the nature and purpose of the study. A positive answer signaled the respondent’s agreement to participate in the study. Participation was anonymized and each participant was attributed a code.

## Author Contributions

CG, PG, CB, and JF contributed to the conception and design of the study. CG contributed to the acquisition of the data. PG and CG contributed to data analysis and interpretation of the data. CG and PG drafted the manuscript. CG, PG, CB, and JF were involved in the critical revision of the manuscript.

## Conflict of Interest Statement

The authors declare that the research was conducted in the absence of any commercial or financial relationships that could be construed as a potential conflict of interest.
